# Vancomycin-Resistant *Staphylococcus aureus,* Michigan, USA, 2007

**DOI:** 10.3201/eid1506.081312

**Published:** 2009-06

**Authors:** Jennie Finks, Eden Wells, Teri Lee Dyke, Nasir Husain, Linda Plizga, Renuka Heddurshetti, Melinda Wilkins, James Rudrik, Jeffrey Hageman, Jean Patel, Corinne Miller

**Affiliations:** Michigan Department of Community Health, Lansing, Michigan, USA (J. Finks, E. Wells, T.L. Dyke, M. Wilkins, J. Rudrik, C. Miller); Centers for Disease Control and Prevention, Atlanta, Georgia, USA (J. Finks, J. Hageman, J. Patel); St. John Macomb-Oakland Hospital, Warren, Michigan, USA (N. Husain, L. Plizga); William Beaumont-Troy Hospital, Troy, Michigan, USA (R. Heddurshetti)

**Keywords:** VRSA, MRSA, Staphylococcus aureus, antimicrobial resistance, vancomycin resistance, bacteria, Michigan, USA, dispatch

## Abstract

Vancomycin-resistant *Staphylococcus aureus* (VRSA) infections, which are always methicillin-resistant, are a rare but serious public health concern. We examined 2 cases in Michigan in 2007. Both patients had underlying illnesses. Isolates were *vanA*-positive. VRSA was neither transmitted to or from another known VRSA patient nor transmitted from patients to identified contacts.

Vancomycin continues to be used as a first-line antimicrobial agent for the treatment of infection with methicillin-resistant *Staphylococcus aureus* (MRSA). Because alternative treatments are limited, development of resistance to vancomycin can make treatment of MRSA infections increasingly difficult. Fortunately, only 7 cases of vancomycin-resistant *S. aureus* (VRSA) infection, which is always methicillin-resistant, have been reported in the United States (Table) (*1*); 5 of these cases occurred in Michigan. We report 2 additional cases of VRSA that occurred in Michigan in 2007. The Michigan Department of Community Health (MDCH) examined the patients’ records, compared genetic characteristics of isolates, assessed possible transmission to contacts, and assessed infection control practices at facilities providing patient care.

**Table:** Vancomycin-resistant *Staphylococcus aureus* isolates detected in the United States, 2002–2006

Isolate no.	State	Date isolated
1	Michigan	2002 Jun
2	Pennsylvania	2002 Sep
3	New York	2004 Mar
4	Michigan	2005 Feb
5	Michigan	2005 Oct
6	Michigan	2005 Dec
7	Michigan	2006 Oct

## The Cases

From each patient’s medical records, we collected information about demographics and concurrent illness, antimicrobial drug history, history of prior MRSA and vancomycin-resistant *Enterococcus* spp. (VRE) infections, and VRSA site co-infections. Initial isolate identification and antimicrobial drug susceptibility testing were conducted by 2 independent Michigan hospitals. Confirmatory organism identification by conventional biochemical methods and antimicrobial drug susceptibility testing were performed by MDCH’s Bureau of Laboratories (*2*,*3*). Vancomycin resistance is defined as MIC >16 µg/mL (*4*). Isolates were submitted to the Centers for Disease Control and Prevention (CDC) for PCR testing for *van* genes, which encode vancomycin resistance, and for genetic analysis by pulsed-field gel electrophoresis (PFGE) and plasmid restriction digest to compare with other VRSA isolates (*5*–*7*).

By following the CDC guide for investigating and controlling VRSA (*8*), we defined periods of potential transmissibility. The length of this period is flexible: start date depends on recent culture results, patient care settings, and clinical assessment; end date is determined by 2 negative cultures, which are submitted weekly posttherapy. To develop a list of potential patient contacts, we assessed healthcare visits, community activities, and personal acquaintances from this period. Contacts were then screened for VRSA, starting with persons who had had the most extensive contact (*8*). Swabs of bilateral anterior nares and open wounds were collected from each contact and spread onto blood agar (TSA with sheep blood) and mannitol salt agar (both from Remel, Lenexa, KS, USA). Plates were incubated for 72 h at 35°C and then for 72 h at room temperature; results were reported as negative when no growth occurred after incubation at these conditions. Serial swabs were collected from contacts who had ongoing exposure. Infection control practices were assessed at all facilities that had provided care to each patient.

**Figure 1:**
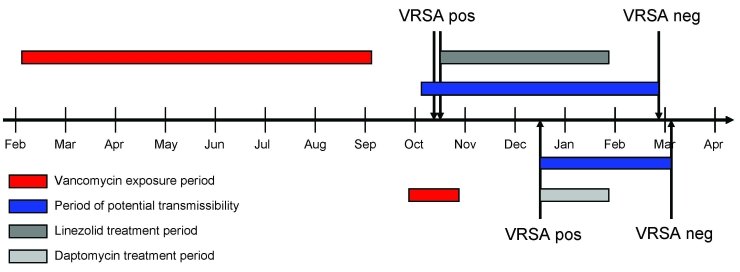
Vancomycin-resistant *Staphylococcus aureus* (VRSA) culture, treatment, and period of potential transmissibility timelines, 2 patients, February 2007–April 2008. Top, patient 1; bottom, patient 2; pos, positive; neg, negative.

On October 12, 2007, VRSA and MRSA were cultured from a right plantar foot wound of a 48-year-old patient (patient 1) who had a history of insulin-dependent diabetes, chronic foot ulcers, and prior concurrent MRSA and VRE infections. The patient had recently received vancomycin and ceftriaxone for 7 months to treat osteomyelitis of the right metatarsals. The patient’s VRSA infection was treated with linezolid and meropenem for 15 weeks. Final VRSA-negative posttreatment swabs were collected on February 26, 2008. The investigation was closed 134 days after initial VRSA-positive culture (Figure 1).

On December 13, 2007, VRSA, VRE, and *Citrobacter youngae* were cultured from a left plantar foot wound of a 54-year-old patient (patient 2) who had inadequately controlled insulin-dependent diabetes. This patient had no documented history of MRSA infection and had recently received vancomycin and levofloxacin for 4 weeks to treat osteomyelitis of the left metatarsals. The patient’s VRSA infection was treated with daptomycin for 6 weeks. Final VRSA-negative posttreatment swabs were collected on March 4, 2008. The investigation was closed 81 days after initial VRSA-positive culture (Figure 1).

**Figure 2:**
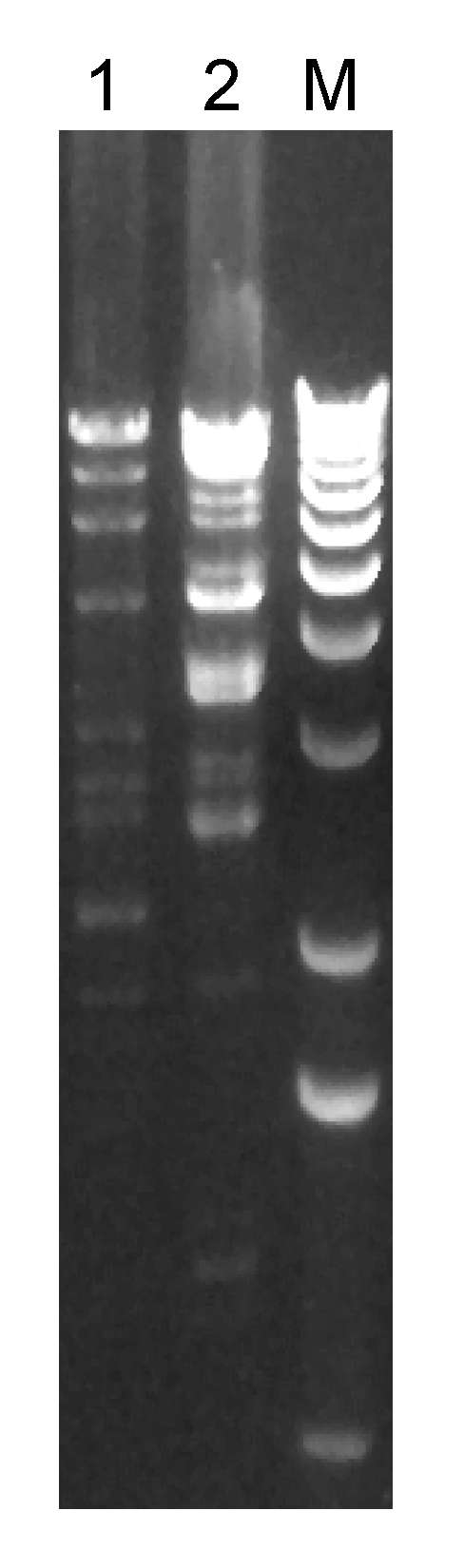
Restriction enzyme (*Hind*III) digest of plasmids prepared from vancomycin-resistant *Staphylococcus aureus* (VRSA) isolates from 2 patients in Michigan, USA, 2007. Each lane is labeled with the VRSA isolate number; lane M, 1-kb molecular marker.

The VRSA isolates from each patient were highly resistant to vancomycin (each MIC 1,024 µg/mL) but susceptible to daptomycin, linezolid, quinupristin/dalfopristin, rifampin, tetracycline, and tigecycline. The isolate from patient 1 was additionally susceptible to chloramphenicol. Isolates from both patients were resistant to trimethoprim/sulfamethoxazole, whereas the 7 VRSA isolates tested previously had been susceptible. VRSA isolates from the 2 patients were PCR positive for the *vanA* gene, 1 of the 7 *van* genes that encode vancomycin resistance. PFGE results for both isolates differed from all other US VRSA isolates (data not shown). VRSA isolates from the 2 patients reported here had distinct plasmids (Figure 2), and plasmid restriction patterns of these isolates differed from the other 7 US VRSA isolates (*7*).

The period of potential transmissibility for patient 1 began October 5, a total of 7 days before the date of positive culture, because of possible exposures during a recent hospitalization; the period ended February 26. The period of potential transmissibility for patient 2 began December 13, the date of positive culture, and ended March 4 (Figure 1). Contacts for patient 1 were evaluated at 7 locations and for patient 2 at 5 locations. For patient 1, a total of 111 swabs were collected from 75 (99%) of 76 identified contacts; 19 (25%) contacts were positive for *S. aureus*; 5 (7%) were positive for MRSA**.** For patient 2, a total of 140 swabs were collected from 126 (98%) of 128 identified contacts; 40 (32%) contacts were positive for *S. aureus,* 13 (10%) were positive for MRSA. No contacts of either patient were positive for VRSA. No infection control breaches were identified.

## Conclusions

These 2 recent cases are consistent with cases reported in the review by Sievert et al. (*1*): each patient had substantial underlying concurrent conditions that contributed to the illnesses, genetic analysis of these isolates indicates that VRSA was not transmitted to or from another known VRSA patient, and no identified transmission occurred from patients to contacts. Also consistent with most previous cases, each patient reported here had a history of VRE and of vancomycin use <3 months before VRSA infection. However, patient 2 did not have a documented history of MRSA infection or colonization. Given the patient’s history of diabetes and chronic foot wounds, MRSA might have been present but undiagnosed.

Data from the other 7 US cases support the hypothesis that patients at risk for VRSA are co-infected or co-colonized with VRE and MRSA, which enables transfer of the *vanA* gene from VRE to MRSA in a biofilm environment, resulting in a VRSA strain. Despite attempts, only 1 laboratory has reported in vitro transfer of vancomycin resistance from VRE to *S. aureus*, demonstrating that interspecies transfer is not frequent (*9*). However, in vitro transfer of vancomycin resistance from VRSA to *S. aureus* has been demonstrated, reinforcing concerns about potential intraspecies transfer of vancomycin resistance among staphylococci (*10*).

Although VRSA infection continues to be rare and no transmission has been identified, it remains a serious public health concern, especially in Michigan where 7 of the 9 US cases have occurred. MDCH continues to educate healthcare providers about correct infection control strategies (*11*) and prudent antimicrobial drug use. MDCH's Bureau of Laboratories provides guidance to hospitals on methods of VRSA detection. MDCH field staff educate patients and their household contacts about wound care, hand and personal hygiene, and the importance of regular monitoring and control of diabetes, a common underlying condition with VRSA infection. Despite these efforts, questions remain unanswered, including why 7 of the 9 US VRSA cases occurred in Michigan. Before targeted prevention strategies can be developed, more research is needed to improve understanding of the microbiologic, clinical, and epidemiologic risk factors for VRSA.
